# Prevalence of lumbar spondylolysis and spondylolisthesis in patients with degenerative spinal disease

**DOI:** 10.1038/s41598-020-63784-0

**Published:** 2020-04-21

**Authors:** Yasuchika Aoki, Hiroshi Takahashi, Arata Nakajima, Go Kubota, Atsuya Watanabe, Takayuki Nakajima, Yawara Eguchi, Sumihisa Orita, Hiroyuki Fukuchi, Noriyuki Yanagawa, Koichi Nakagawa, Seiji Ohtori

**Affiliations:** 1 Department of Orthopaedic Surgery, Eastern Chiba Medical Center, Togane, Japan; 2 Department of Orthopaedic Surgery, Toho University Sakura Medical Center, Sakura, Japan; 3 Department of Orthopaedic Surgery, Graduate School of Medicine, Chiba University, Chiba, Japan; 4 Department of General Medical Science, Graduate School of Medicine, Chiba University, Chiba, Japan; 5 Department of Radiology, Eastern Chiba Medical Center, Togane, Japan

**Keywords:** Musculoskeletal abnormalities, Bone

## Abstract

Lumbar spondylolysis generally occurs in adolescent athletes. Bony union can be expected with conservative treatment, however, the fracture does not heal in some cases. When the fracture becomes a pseudoarthrosis, spondylolysis patients have the potential to develop isthmic spondylolisthesis. A cross-sectional study was performed to determine the incidence of spondylolysis and spondylolisthesis, and to elucidate when and how often spondylolisthesis occurs in patients with or without spondylolysis. Patients undergoing computed tomography (CT) scans of abdominal or lumbar regions for reasons other than low back pain were included (n = 580). Reconstruction CT images were obtained, and the prevalence of spondylolysis and spondylolisthesis were evaluated. Of the 580 patients, 37 patients (6.4%) had spondylolysis. Of these 37 patients, 19 patients (51.4%) showed spondylolisthesis, whereas only 7.4% of non-spondylolysis patients showed spondylolisthesis (p < 0.05). When excluding unilateral spondylolysis, 90% (18/20) of spondylolysis patients aged ≥60 years-old showed spondylolisthesis. None of the patients with isthmic spondylolisthesis had received fusion surgery, suggesting that most of these patients didn’t have a severe disability requiring surgical treatment. Our results showed that the majority of bilateral spondylolysis patients aged ≥60 years-old show spondylolisthesis, and suggest that spondylolisthesis occurs very frequently and may develop at a younger age when spondylolysis exists.

## Introduction

Lumbar spondylolysis, which is considered a stress fracture of the pars interarticularis, commonly occurs in adolescent patients^1^. In adolescent patients with acute lumbar spondylolysis, bony union can be expected with adequate conservative treatment, such as wearing a brace and refraining from sports activity^2,3^; however, in some cases, the fracture does not heal and it becomes a pseudoarthrosis.

At this moment, there is little information about the long-term prognosis of lumbar spondylolysis in cases where the defect becomes a pseudoarthrosis. Thus, patients may have justified anxiety whether they develop severe lumbar degenerative disease. To clarify the actual long-term prognosis of spondylolysis, a prospective study should be performed. However, because spondylolysis patients are usually adolescent, it is not easy to perform a prospective study requiring over fifty or sixty-year follow-up. We propose that patients need information about the future possibility of lumbar degenerative disease because of the presence of spondylolysis suggested by retrospective or cross-sectional studies.

The purpose of the present study is to clarify the future possibility of lumbar spondylolisthesis in patients with lumbar spondylolysis. Although previous studies demonstrated that spondylolysis patients have higher future incidences of disc degeneration and spondylolisthesis^4–7^, there is no detailed information regarding when and how often spondylolisthesis occurs in patients with or without spondylolysis. Therefore, we examined the age-specific prevalence of spondylolysis and spondylolisthesis in non-low back pain (LBP) patients.

## Materials and Methods

Consecutive patients undergoing computed tomography (CT) scans of abdominal or lumbar regions between July 2015 and January 2016 in our institution were included. Only patients undergoing CT scan for reasons other than low back disorders were included (n = 580). The study was carried out in accordance with the Declaration of Helsinki, and the study protocol was approved by the Institutional Review Board of Eastern Chiba Medical Center, and all patients had signed the informed consent.

Patients’ age and sex were reviewed, and sagittal multiplanar reconstruction CT images were obtained and evaluated by two independent observers (spine surgeons). If their opinions differed, the final description was determined by a third observer. First, the presence of spondylolysis was examined, and whether it was unilateral or bilateral was evaluated. Second, the presence of vertebral slip (>3 mm) was examined in patients with or without spondylolysis. Finally, levels, sex- and age-specific prevalence of spondylolysis and spondylolisthesis were examined.

### Data analysis

To compare the prevalence of spondylolysis and spondylolisthesis, the chi-square test was used. A *p* value < 0.05 was considered to be statistically significant.

## Results

### The prevalence of spondylolysis

Of the 580 general population patients (mean age: 64.4 ± 18.9 years-old; 336 male/244 female, Table 1), 37 patients (6.4%; 26 male and 11 female) had spondylolysis (Tables 1 and 2). One patient had spondylolysis at 2 levels. Levels of spondylolysis were 1 at L1, 3 at L4 and 34 at the L5 vertebrae. As shown in Table 2, male showed higher incidence of spondylolysis (7.7%: 26/336 patients) than female (4.5%: 11/244 patients), although a significant difference was not observed (Table 2). Five of 37 patients had unilateral spondylolysis, and the remaining 32 patients had bilateral spondylolysis (Table 2).

**Table 1:** Characteristics of patients undergoing computed tomography (n = 580).

Age	(years)	64.4 ± 18.9 (9–95)
Sex	(M/F)	336 / 244
Spondylolysis	(%)	6.38% (37cases)

M = male, F = female.

**Table 2:** The prevalence of spondylolysis in male and female.

	Male	Female	Total
% of spondylolysis	7.7% (26/336)	4.5% (11/244)	6.4% (37/580)
Unilateral: Bilateral	4: 22	1: 10	5: 32

### The prevalence of spondylolisthesis

Of the 37 patients with spondylolysis, 19 (51.4%) patients showed spondylolisthesis (Table 3). None of the five patients with unilateral spondylolysis showed spondylolisthesis. Of the remaining 32 patients with bilateral spondylolysis, the incidence of spondylolisthesis was 59.4%. Because the defect of pars interarticularis causes instability between the vertebra with spondylolysis and one level caudal to that vertebra, all spondylolisthesis events occurred at one level caudal to the spondylolysis, except one case showing spondylolisthesis at one level cranial to the spondylolysis. Of the 19 spondylolysis patients showing spondylolisthesis, 7 patients (36.8%) had Meyerding grade 2 spondylolisthesis; however, no patient showed spondylolisthesis greater than Meyerding grade 2.

**Table 3:** The prevalence of spondylolisthesis in patients with or without spondylolysis (lysis).

	Total		Male		Female	
Age (yr)	Lysis (+)	Lysis (−)	Lysis (+)	Lysis (−)	Lysis (+)	Lysis (−)
−49	0/9 (2)	0/113	0/4 (1)	0/70	0/5 (1)	0/43
50–59	1/5	1/65	1/5	1/45	0/0	0/20
60–69	5/5	7/117	4/4	1/66	1/1	6/51
70–79	4/7 (2)	14/118	2/5 (2)	4/66	2/2	10/52
80-	9/11 (1)	18/130	6/8 (1)	7/63	3/3	11/67
Total	19/37 (5)	40/543	13/26 (4)	13/310*	6/11 (1)	27/233*

Parenthesis indicates number of unilateral spondylolysis.

*Significant difference between male and female.

In patients showing no spondylolysis, a significantly lower prevalence of spondylolisthesis (7.4%: 40/543 patients, p < 0.001) was observed when compared with patients with spondylolysis. None of the patients showed vertebral slip greater than Meyerding grade I. The prevalence of Meyerding grade spondylolisthesis was significantly higher in spondylolysis patients (7/19) than in non-spondylolysis patients (0/40, p < 0.001). Female showed significantly higher prevalence of spondylolisthesis (11.6%) than male (4.2%) in patients without spondylolysis (Table 3). None of the patients with isthmic spondylolisthesis had received fusion surgery, suggesting that most of these patients did not have a severe disability requiring surgical treatment.

### The age-specific prevalence of spondylolisthesis with or without spondylolysis

In both patient groups with or without spondylolysis, no patient younger than 50 years-old had spondylolisthesis. The prevalence of spondylolisthesis showed an age-dependent increase in spondylolysis patients, as well as in non-spondylolysis patients (Table 3). When excluding unilateral spondylolysis patients, the majority of spondylolysis patients (90%; 18/20) aged ≥60 years-old showed spondylolisthesis, whereas only 8.3% (1/12) of patients ˂60 years-old showed spondylolisthesis (p = 0.02). In the non-spondylolysis patients, the prevalence of spondylolisthesis in patients ≥60 years-old (10.7%: 39/365) was significantly lower than in spondylolysis patients ≥60 years-old (78.3%: 18/23, p < 0.001).

## Discussion

Our results suggest that an extremely high percentage of spondylolysis patients over 60 years-old have spondylolisthesis at one level caudal to the spondylolysis, particularly if they have bilateral spondylolysis. The prevalence of spondylolysis differs depending on ethnicity and sex. Sakai *et al*. reported that the prevalence of spondylolysis in the Japanese general population is 5.9%, and that male (7.9%) showed a higher prevalence than female (3.9%)^8^. In our study, we confirmed that the ethnicity of all patients seems to be Japanese, or at least, east Asia, and showed a prevalence similar to the previous study, suggesting the data of our study may reflect the general population in Japan. The study of Sakai and others^8^, as well as our study, showed that unilateral spondylolysis rarely showed spondylolisthesis, whereas bilateral spondylolysis often showed spondylolisthesis. These results suggest that the prognosis of unilateral spondylolysis is more favorable than that of bilateral spondylolysis. Lemoine *et al*. reported that the prevalence of bilateral spondylolysis increased after children have learned to walk^9^. However, Brooks *et al*. reported that the prevalence of lumbar spondylolysis does not increase in patients older than 20 years^10^. Therefore, it is generally recognized that spondylolysis occurs in adolescent under the age of 20 years. In contrast, the prevalence of spondylolisthesis would increase depending on age^11,12^. Thus, to clarify the actual prevalence of spondylolisthesis, only elderly patients should be included in the analysis. Our results suggest that the prevalence of spondylolysis patients showing spondylolisthesis rises at the ages of 60–69. When limited to patients over 60 years-old, the incidence of spondylolisthesis was extremely high (90%: 18/20) in bilateral spondylolysis patients. On the other hand, the incidence of spondylolisthesis in non-spondylolysis patients was only 10.7% (39/365) even when limited to patients over 60 years-old. These results indicate that bilateral spondylolysis is strongly correlated with the prevalence of spondylolisthesis. Our results showed that spondylolysis patients more often showed Meyerding grade 2 spondylolisthesis. From these observations, it can be seen that spondylolisthesis develops more often, and more severely in patients with spondylolysis, when compared with patients without spondylolysis.

Brinjikji *et al*. reported that spondylolysis is more prevalent in young adults (<50 years-old) with back pain compared with asymptomatic individuals^13^, suggesting that spondylolysis patients may have a greater chance to have LBP during middle age. Spondylolysis patients often develop spondylolisthesis in their old age, and a certain number of patients may have severe LBP or radicular symptoms^14^. This study has several limitations. First, the patients visited our hospital because they had a disease and underwent CT scans for various reasons, including polytrauma, gastrointestinal symptoms, detailed examination for liver dysfunction, etc. We could not exclude the possibility that their disease influenced the prevalence of spondylolysis and spondylolisthesis. Second, we did not have precise information about whether they had LBP and did not perform a follow-up study. However, this study may help us to understand the prognosis of spondylosis to some extent.

To clarify the long-term prognosis of spondylolysis more accurately, a prospective study should be performed. However, it takes a very long time to get results from prospective studies because spondylolysis usually occurs in patients younger than 20 years old^9,10^. Consequently, we have little current information about the long-term prognosis of spondylolysis elucidated by prospective studies. We, therefore, suggest an estimated prognosis that the majority of bilateral spondylolysis patients over 60 years-old develop spondylolisthesis from the results of our cross-sectional study. The results of our study provide information suggesting the long-term prognosis of spondylolysis, and are helpful for spondylolysis patients as well as physicians to make treatment decisions.
